# Choice of observational study design impacts on measurement of antipsychotic risks in the elderly: a systematic review

**DOI:** 10.1186/1471-2288-12-72

**Published:** 2012-06-08

**Authors:** Nicole Pratt, Elizabeth E Roughead, Amy Salter, Philip Ryan

**Affiliations:** 1 Quality Use of Medicines and Pharmacy Research Centre; Sansom Institute, University of South Australia, Adelaide, Australia; 2 Discipline of Public Health, School of Population Health and Clinical Practice, University of Adelaide, Adelaide, Australia

**Keywords:** Antipsychotics, Review, Death, Cerebrovascular events, Stroke, Hip fracture, Pneumonia, Hospitalisation

## Abstract

**Background:**

Antipsychotics are frequently and increasingly prescribed to treat the behavioural symptoms associated with dementia despite their modest efficacy. Evidence regarding the potential adverse events of antipsychotics is limited and little is known about the longer-term safety of these medicines in the elderly. The aim of this review was to determine the impact of the choice of observational study design and methods used to control for confounding on the measurement of antipsychotic risks in elderly patients.

**Methods:**

We searched PUBMED and the Cochrane controlled trials register for double-blind randomised controlled trials (RCTs), meta-analyses and published observational studies of antipsychotics.

**Results:**

Forty four studies were identified for the endpoints; death, cerebrovascular events, hip fracture and pneumonia. RCTs found a 20% to 30% increased risk of death, or an absolute increase of 1extra death per 100 patients with atypical antipsychotics compared to non-use. Cohort and instrumental variable analyses estimated between 2 to 7 extra deaths per 100 patients with conventional compared to atypical antipsychotics. RCTs found a 2 to 3 times increased risk of all cerebrovascular events with atypical antipsychotics compared to placebo and no association with serious stroke that required hospitalisation. Observational studies using cohort and self-controlled case-series designs reported similar results; no association where the endpoint was stroke causing hospitalisation and a doubling of risk when minor stroke was included. No RCTs were available for the outcome of hip fracture or pneumonia. Observational studies reported a 20% to 40% increased risk of hip fracture with both antipsychotic classes compared to non-use. The risk of pneumonia was a 2 to 3 times greater with both classes compared to non-use while a self-controlled case-series study estimated a 60% increased risk. Conventional antipsychotics were associated with a 50% greater hip fracture risk than atypical antipsychotics, while the risk of pneumonia was similar between the classes.

**Conclusions:**

Choice of observational study design is critical in studying the adverse effects of antispychotics. Cohort and instrumental variable analyses gave more consistent results to clinical studies for mortality outcomes as have self-controlled case-series for the risk of cerebrovascular events and stroke. Observational evidence has highlighted the potential for antipsychotics to be associated with serious adverse events that were not reported in RCTs including hip fracture and pneumonia. Good quality observational studies are required, that employ appropriate study designs that are robust towards unmeasured confounding, to confirm the potential excess risk of hip fracture and pneumonia with antipsychotics.

## Background

Antipsychotics are frequently, and increasingly, prescribed to treat the behavioural symptoms associated with dementia despite their modest efficacy and potential for serious side effects [1,2]. A Cochrane review [2] failed to find any evidence of benefit with haloperidol treatment in patients with agitated dementia and recommended that it should not be used routinely. Another Cochrane review [1] found that atypical antipsychotics may help to improve symptoms of dementia such as aggression, psychosis and agitation,[3-7] however, improvements were often limited to patients with more severe dementia [5]. Large head to head randomised trials of atypical and conventional antipsychotics have not been performed in the elderly, however, one post-hoc analysis of a small experimental study [8] found that risperidone may be more effective in reducing aggressiveness than haloperidol.

Side effects are common with both classes of antipsychotics and include; extra-pyramidal symptoms [3-7] and somnolence [4-6]. These effects may be less frequent [9] and less severe [8] with risperidone compared to haloperidol, but only at lower doses of the medicines [9]. Experimental evidence regarding the more serious adverse events of antipsychotics is limited to atypical antipsychotics and little is known about the safety of conventional antipsychotics in the elderly. Furthermore, most randomised controlled trials have limited follow-up of up to 12 weeks [1] and the safety of both antipsychotic classes with long-term treatment remains unclear.

Post marketing observational studies of antipsychotics have identified many safety issues associated with antipsychotics, however, study results vary and conclusions are inconsistent.

Previous reviews of observational studies of antispychotics have focused on the clinical safety of conventional and atypical antipsychotics [10-13]. The aim of this review was to synthesise the current evidence from observational studies, regarding the serious adverse events of antipsychotics in elderly patients and to determine the impact of the observational study design utilised and the technique employed to control for confounding on study results.

A review of available meta-analyses of randomised controlled trial evidence was performed for comparison purposes. The endpoints of interest were death, cerebrovascular events, hip fracture and pneumonia.

## Methods

This review was performed according to the Quality of Reporting of Meta-analyses (PRISMA http://www.prisma-statement.org/) guidelines (see online Additional File 1 PRISMA 2009 checklist).

We searched the PUBMED database and the Cochrane controlled trials register for all English-language articles published up to December 2010. We also conducted a manual search of bibliographies for other relevant articles. We included double-blind randomised controlled trials (RCTs), meta-analyses and published observational studies that evaluated adverse events of either conventional or atypical antipsychotic medications in elderly populations. All studies were included if they reported at least one of the adverse events of interest; death, cerebrovascular events, hip fracture or pneumonia. Studies specifically investigating the use of antipsychotics in schizophrenic patients were not included. Individual RCTs contributing to meta-analysis were not included in this review to avoid double counting of studies. In PUBMED we combined the results of 2 search domains: Dementia (MESH terms Dementia OR Dementia, Vascular NOT Schizophrenia), and drug therapy (Antipsychotic Agents) with each of the following searches for the outcomes of interest; death (Death OR Death, Sudden, Cardiac OR Death, Sudden OR Mortality), cerebrovascular events (Stroke), hip fracture (Hip Fractures) and pneumonia (Pneumonia OR Pneumonia, Bacterial OR Pneumonia, Aspiration). Author 1 (NP) was responsible for the retrieval and review of studies to be included in the final review. Author 2 (ER) provided independent assessment of studies when required.

**Figure 1:**
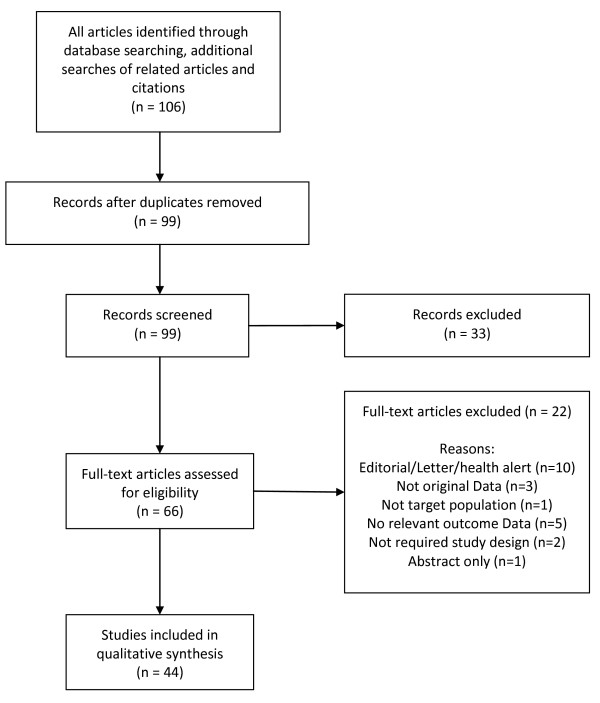
Flow diagram of the selected studies.

We identified 106 articles, 7 of which were duplicates. Of the 99 unique records, 33 articles were excluded based upon review of the title and abstract. We reviewed 66 full text articles, of which 22 were excluded (Figure1). Articles were excluded if they were editorials, letters, did not contain original data or outcome data of interest or were commentaries on already included published data.

We included observational studies that employed either a case–control design, a cohort design or a self-controlled case-series design. Studies that also used an instrumental variable analysis in addition to the standard cohort analysis were also included. The use of instrumental variables has been suggested as a possible alternative to conventional analyses when there is concern about the effect of unmeasured confounding [14,15]. Instrumental variable (IV) analysis attempts to mimic the process of randomisation in an RCT by exploiting the existence of another variable (the instrument) which can be measured in the available data, which is highly correlated with the probability of exposure but unrelated to the outcome of interest except through its association with treatment [16]. The instrument is similar to random arm assignment in that it should distribute both measured and unmeasured patient characteristics evenly between exposure groups resulting in an estimate less affected by confounding. The self-controlled case-series design compares the risk of adverse events in periods of exposure compared to non-exposure within the same person. This design attempts to limit the effects of major unmeasured confounders as the within-person study design controls implicitly for confounders that do not vary over time [17].

The final identified studies were grouped by outcome type and categorised according to the primary study medication comparison. Studies were rated according to a hierarchy of evidence of study designs [18] with meta analyses considered as the highest level of evidence, observational studies were considered as lower quality. Data were extracted including the study population, study size, study design, propensity for bias, procedures employed to minimise bias, a rating of study quality, duration of follow-up, outcome rate in the reference group, effect estimate (either rate ratio, odds ratio, hazard ratio or risk difference depending on the study design utilised) and appropriate 95% confidence intervals. The propensity for bias was assigned as ‘Low’ for RCTs and ‘Moderate’ for observational studies. Study quality was assigned as ‘High’ for RCTs. Observational studies were assigned a ‘Moderate’ quality rating if specific methods were employed to minimise bias otherwise studies were assigned a ‘Low’ quality if they made only minimal efforts to control for potential bias. Study designs such as the self-controlled case-series or instrumental variable analysis are not formally recognised in the hierarchy of evidence, however, we have considered these studies as they attempt to account for the common problem of unmeasured confounding in observational studies. The probable place of instrumental variable analysis in the hierarchy is either equivalent to or better than cohort studies. The place of the self-controlled case-series is as yet unclear.

In this review we specifically aimed to determine the extent to which the study design employed and the methods utilised to control for confounding, impacted on study results. Thus it was considered inappropriate to proceed with a formal meta-analysis due to the likely heterogeneity of studies.

## Results

This review included 44 studies, 16 evaluated the risk of death, 18 evaluated cerebrovascular events, 8 evaluated the risk of hip fracture and 6 evaluated the risk of pneumonia associated with antipsychotic prescribing. Details of the studies meeting our search criteria are presented in Table 1 for death outcomes, Table 2 for cerebrovasular events, Table 3 for hip fracture and Table 4 for pneumonia.

**Table 1:** Studies on the risk of death associated with antipsychotic medicines

**Study**	**Population**	**Outcome**	**Propensity for bias/Study quality**	**Procedures to minimize bias**	**Follow-up**	**Outcome rate in reference group**	**Result**
***LEVEL I Evidence: Meta-Analyses***							
***Studies that compared atypical antipsychotic (ATYP) treatment to placebo (PLA)***							
Katz [5]	895 Institutionalised dementia patients (age>=55)	Death	Low/High	Yes	12 weeks	1.8%	HR(RISP v PLA); 1.26 95% CI; 0.53-2.99
Schneider [19]	5,204 Dementia patients (age>55)	Death	Low/High	Yes	6-26 weeks	2.3%	OR(ATYP v PLA); 1.54 95% CI; 1.06-2.23 RD(ATYP - PLA); 0.01 95% CI; 0.004-0.02
						2.8%	OR(RISP v PLA); 1.30 95%CI; 0.76-2.23
Haupt [20]	1,721 Alzheimers Patients (mean age 82.3)	Death	Low/High	Yes	4-12 weeks	3.1%	RR(RISP v PLA); 1.21 95% CI; 0.71-2.06
***LEVEL II Evidence: Randomised Controlled Trial***							
***Studies that compared atypical antipsychotic (ATYP) treatment to placebo (PLA)***							
Ballard [21]	165 Institutionalised dementia patients (mean age 85)	Death	Low/High	Yes	12 months	33%	HR(RISP v PLA); 0.58 95% CI; 0.36-0.92
***Studies that compared conventional antipsychotic (C) treatment to placebo (PLA)***							
DeDeyn [8]	344 Dementia patients (mean age 81 placebo, 82 haloperidol)	Death	Low/High	Yes	12 weeks	3.8%	OR(HAL v PLA); 1.68 95% CI; 0.72-3.92 [19]
***LEVEL III Evidence: Observational Studies***							
***Studies that compared atypical antipsychotic (ATYP) treatment to non-use (NU)***							
***COHORT STUDIES***							
Gill [22]	9,100 matched pairs, Non-institutionalised dementia patients in a universal health fund in Ontario Canada (age>=65)	Death	Moderate/Moderate	Yes (Propensity score matching, sensitivity analysis)	180 days	8.0%	HR(ATYP v NU);1.32 95% CI; 1.12-1.54 RD(ATYP - NU); 1.1 per 100 95% CI; 0.1-2.1
Gill [22]	4,036 matched pairs, Institutionalised dementia patients in a universal health fund in Ontario Canada (age>=65)	Death	Moderate/Moderate	Yes (Propensity score matching, sensitivity analysis)	180 days	15.1%	HR(ATYP v NU);1.23 95% CI; 1.05-1.45 RD(ATYP - NU); 1.5 per 100 95% CI; -0.5-3.4
***CASE-CONTROL STUDIES***							
Trifiro [23]	398 cases, 4,023 controls, dementia patients, Integrated Primary Care Information Database (Netherlands)(age>85)	Death	Moderate/Low	Yes (matching on age and duration of dementia)	9 years	NA	OR(ATYP v NU); 2.2 95% CI; 1.2-3.9
Raivio [24]	254 institutionalized dementia patients (Finland) (age>70)	Death	Moderate/Low	Yes (covariate adjustment)	2 years	49.6%	HR(ATYP v NU);0.49 95% CI; 0.24-0.99
***Studies that compared conventional antipsychotic (CONV) treatment to non-use (NU)***							
***COHORT STUDIES***							
Ray [25]	1,282,995 Non-institutionalised dementia, medicaid-enrolled patients (Tennessee) (age 15-84)	Sudden cardiac Death	Moderate/Moderate	Yes (covariate adjustment, sensitivity analysis)	1 year	11.3/10000 PY	Moderate Dose >100mg: RR(CONV v NU); 2.39 95% CI; 1.77-3.22 Low Dose <100mg: RR(CONV v NU); 1.3 95% CI; 0.98-1.72
Kales [26]	10,615 Veterans enrolled in VA Serious Mental Illness Treatment Research and Evaluation Centre, Dementia Patients (US) (age>65)	Death	Moderate/Moderate	Yes (Propensity score adjustment, sensitivity analysis, subgroup analysis)	1 year	25.2%	RR(NU v CONV); 0.66 95% CI; 0.53-0.82
***CASE-CONTROL STUDIES***							
Trifiro [23]	398 cases, 4,023 controls, dementia patients Integrated Primary Care Information Database (Netherlands) (age>85)	Death	Moderate/Low	Yes (matching on age and duration of dementia)	9 years	NA	OR(CONV v NU); 1.8 95% CI; 1.4-2.3
Raivio [24]	254 institutionalized dementia patients (Finland) (age>70)	Death	Moderate/Low	Yes (covariate adjustment)	2 years	49.6%	HR(CONV v NU); 0.68 95% CI; 0.46-1.03
***Studies that compared conventional antipsychotic (CONV) and atypical antipsychotic (ATYP) treatment***							
***COHORT STUDIES***							
Gill [22]	9,100 matched pairs, Non-institutionalised dementia patients (age>=65)	Death	Moderate/Moderate	Yes (Propensity score matching, sensitivity analysis)	180 days	10.7%	HR(CONV v ATYP); 1.23 95% CI; 1.00-1.50 RD(CONV - ATYP); 2.6 per 100 95% CI; 0.5-4.5
Gill [22]	4,036 matched pairs, Institutionalised dementia patients (age>=65)	Death	Moderate/Moderate	Yes (Propensity score matching, sensitivity analysis)	180 days	17.8%	HR(CONV v ATYP); 1.27 95% CI; 1.09-1.48 RD(CONV - ATYP); 2.2 per 100 95% CI; 0.0-4.4
Hollis [27]	16,634 Australian Department of Veterans Affairs Veterans/spouses (Australia) (age>65)	Death	Moderate/Moderate	Yes (covariate adjustment)	2 years	246 per 1000	RR (HALO v OLA); 2.26 95% CI; 2.08-2.47 RR (CHL v OLA); 1.39 95% CI; 1.15-1.67
Hollis [27]	6,602 Institutionalised Australian Department of Veterans Affairs Veterans/spouses (Australia) (age>65)	Death	Moderate/Moderate	Yes (covariate adjustment)	2 years	291 per 1000	RR (HALO v OLA); 1.67 95% CI; 1.50-1.84 RR (CHL v OLA); 1.75 95% CI; 1.31-2.34
Kales [26]	10,615 Veterans enrolled in VA Serious Mental Illness Treatment Research and Evaluation Centre, Dementia Patients (US) (age>65)	Death	Moderate/Moderate	Yes (Covariate and propensity score adjustment, sensitivity analysis, subgroup analysis)	1 year	25.2%	Covariate adjusted RR(ATYP v CONV); 0.93 95% CI; 0.75-1.16
Schneeweiss [28]	37,241 British Columbia Residents (Canada) (age>=65)	Death	Moderate/Moderate	Yes (Covariate and propensity score adjustment, instrumental variable analysis)	180 days	9.6%	Covariate adjusted HR(CONV v ATYP); 1.32 95% CI; 1.23-1.42 PS adjusted HR(CONV v ATYP); 1.39 95% CI; 1.30-1.49 IV RD(CONV – ATYP); 4.2 per 100, 95% CI; 1.2-7.3
Wang [29]	22,890 Drug Insurance Beneficiaries (Pennsylvania US) (age>=65)	Death	Moderate/Moderate	Yes (Covariate and propensity score adjustment, instrumental variable analysis)	180 days	14.6%	Covariate adjusted HR(CONV v ATYP); 1.37 95% CI; 1.27-1.49 Propensity score adjusted adjusted HR(CONV v ATYP); 1.37 95% CI; 1.27-1.49 IV RD(CONV - ATYP); 7.3 per 100 95% CI; 2.0-12.6
Liperoti [30]	9,729 Institutionalised dementia patients (age >=65)	Death	Moderate/Moderate	Yes (covariate adjustment, sensitivity analysis, subgroup analysis)	180 days	40.0 per 100 person-years	HR(CONV v ATYP); 1.26 95% CI; 1.13-1.42 HR (HALO v RISP); 1.31 95% CI; 1.13–1.53 HR(Phenothiazines V RISP); 1.17 95% CI; 1.00–1.38 HR(Other Conventional v RISP); 1.32 95% CI; 0.99–1.80
Pratt [31]	7,311 Institutionalised Australian Department of Veterans Affairs Veterans/spouses (Australia) (Age >65)	Death	Moderate/Moderate	Yes (Propensity score adjustment, instrumental variable analysis)	1 year	37.4%	Covariate adjusted RD(CONV v ATYP); 8.5 95% CI; 6.2-10.7 Propensity score adjusted RD(CONV v ATYP); 9.1 95% CI; 6.9-11.4 IV RD(CONV - ATYP); 10.1 per 100 95% CI; 6.6-13.7
***CASE-CONTROL STUDIES***							
Trifiro [23]	398 cases, 4,023 controls, dementia patients Integrated Primary Care Information Database (Netherlands) (age>85)	Death	Moderate/Low	Yes (matching on age and duration of dementia)	Up to 9 years	NA	OR(ATYP v CONV); 1.3 95% CI; 0.7-2.4

**Table 2:** Studies on the risk of cerebrovascular events associated with antipsychotic medicines

**Study**	**Population**	**Outcome**	**Propensity for bias / Study quality**	**Procedures to minimize bias**	**Follow-up**	**Outcome Rate in reference group**	**Result**
***LEVEL I Evidence: Meta-Analyses***							
***Studies that compared atypical antipsychotic (ATYP) treatment to placebo (PLA)***							
Ballard [1]	1954 dementia patients (age> 60)	CV Events	Low/High	Yes	10-13 weeks	1.0%	RR (RISP v PLA); 3.64 95% CI; 1.72-7.69
Schneider [6]	5,110 Dementia patients (mean age 81.2)	CV Events	Low/High	Yes	6-26 weeks	0.9% 1.0%	OR (ATYP v PLA); 2.13 95% CI; 1.20-3.75 OR(RISP v PLA); 3.43 95%CI; 1.60-7.32
DeDeyn [4]	1,155 Institutionalised patients (age>=55)	CV Events	Low/High	Yes	12 weeks	1.6%	Rate in Risperidone group 3.9% RR not reported
Hermann [32]	1,721 Dementia patients (age>=55)	CV Events	Low/High	Yes	12 weeks	1.1%	RR (RISP v PLA); 3.2 95% CI; 1.4-7.2
Katz [5]	895 Institutionalised dementia patients (age>=55)	CV Events	Low/High	Yes	12 weeks	0.8%	Rate in Risperidone group 1.6% RR not reported
DeDeyn [4]	1,155 Institutionalised patients (age>=55)	Serious CV Event requiring hosp.	Low/High	Yes	12 weeks	0.7%	Rate in Risperidone group 1.6% RR not reported
Hermann [32]	1,721 Dementia patients (age>=55)	Serious CV Event requiring hosp.	Low/High	Yes	12 weeks	0.6%	RR (RISP v PLA); 2.3 95% CI; 0.5-10.7
***LEVEL III Evidence: Observational Studies***							
***Studies that compared atypical antipsychotic (ATYP) treatment to non-use (NU)***							
***COHORT STUDIES***							
Sacchett [33]	74,162 All patients, General Practitioner Health Search database (Italy) (age>=65)	Diagnosis of stroke (GPs’ medical records) ICD9: 434.9, 438.0, 342	Moderate/Moderate	Yes (covariate adjustment, subgroup analysis)	3.5 months	12 per 1000py	RR (ATYP v UNEX); 2.46 95% CI; 1.07-5.65
Barnett [34]	14,029 Dementia patients, Veterans Affairs Clients (US) (age>=65)	Hospital Admission for CV event ICD9 435, 437, 430, 432, 433, 434	Moderate/Moderate	Yes (covariate adjustment, sensitivity analysis)	18 months	3.2%	HR (ATYP v NU); 1.20 95% CI; 0.83-1.74
***CASE-CONTROL STUDIES***							
Kolanowski [35]	959 Dementia patients, health care insured on Southeast US (age>70)	Diagnosis of Stroke	Moderate/ Low	Yes (unmatchedcovariate adjustment)	45 days	NA	OR (ATYP v NU); 0.98 95% CI; 0.64-1.52
Liperoti [36]	1130 cases, 3658 controls, institutionalised dementia patients in six states in the US (Ohio, Maine, Illinois, Mississippi, South Dakota, New York) (age>85)	Hospital Admission for CV event ICD9 433.0-434.9 (Ischaemic stroke) 435-435.9 (TIA)	Moderate/ Low	Yes (matching on admitted to same facility for septicemia, UTI)	NR	NA	OR (RISP v NU); 0.87 95% CI; 0.67-1.12 OR (OLA v NU); 1.32 95% CI; 0.83-2.11 OR (Other ATYP v NU); 1.57 95% CI; 0.65-3.82
***SELF-CONTROLLED CASE-SERIES STUDIES***							
Douglas [37]	6790 All patients with incident diagnosis of stroke General Practice Research Database (UK) (no age restriction)	Diagnosis of stroke (GPs’ medical records, excluding TIA)	Moderate/Moderate	Yes (within patient design)	NR	NA	IRR (ATYP v NU); 2.32 95% CI; 1.73-3.10
Pratt [38]	10638 Australian Department of Veterans Affairs Veterans/spouses with hospitalization for stroke (Australia) (age>=65)	Hospitalis-ation for stroke ICD-10: I60-I64	Moderate/Moderate	Yes (within patient design)	4 years	NA	IRR (ATYP v NU); 0.9 95% CI; 0.7-1.0
***Studies that compared conventional antipsychotic (CONV) treatment to non-use (NU)***							
***COHORT STUDIES***							
Sacchetti [33]	74,162 All patients, General Practitioner Health Search database (Italy) (age>=65)	Diagnosis of stroke (GPs’ medical records) ICD9: 434.9, 438.0, 342	Moderate/Moderate	Yes (covariate adjustment, subgroup analysis)	3.5 months	12 per 1000py	RR (BUTY v UNEX); 3.55 95% CI; 1.56-8.07 RR (PHENO v UNEX); 5.79 95% CI; 3.07-10.9
Barnett [34]	14,029 Dementia patients, Veterans Affairs Clients (US) (age>=65)	Hospital Admission for CV event ICD9 435, 437, 430, 432, 433, 434	Moderate/Moderate	Yes (covariate adjustment, sensitivity analysis)	18 months	3.2%	HR (CONV v NU); 1.20 95% CI; 0.48-3.47
***CASE-CONTROL STUDIES***							
Kolanowski [35]	959 Dementia patients, health care insured on Southeast US (age>70)	Diagnosis of Stroke	Moderate/ Low	Yes (Unmatched, covariate adjustment)	45 days	NA	OR (CONV v NU); 1.18 95% CI; 0.63-2.24
Liperoti [36]	1130 cases, 3658 controls, institutionalised dementia patients in six states in the US (Ohio, Maine, Illinois, Mississippi, South Dakota, New York) (age>85)	Hospital Admission for CV event ICD9 433.0-434.9 (Ischaemic stroke) 435-435.9 (TIA)	Moderate /Low	Yes (matched; on admitted to same facility for septicemia, UTI)	NR	NA	OR (CONV v NU); 1.24 95% CI; 0.95-1.63
***SELF-CONTROLLED CASE-SERIES STUDIES***							
Douglas [37]	6790 All patients with incident diagnosis of stroke General Practice Research Database (UK) (no age restriction)	Diagnosis of stroke (GPs’ medical records, excluding TIA)	Moderate/Moderate	Yes (within patient design)	NR	NA	IRR (CONV v NU); 1.60 95% CI; 1.55-1.84
Pratt [38]	10638 Australian Department of Veterans Affairs Veterans/spouses with hospitalization for stroke (Australia) (age>=65)	Hospitalis-ation for stroke ICD-10: I60-I64	Moderate/Moderate	Yes (within patient design)	4 years	NA	IRR (CONV v NU); 1.0 95% CI; 0.8-1.2
***Studies that compared all antipsychotics (ANTIP) with no treatment (NU)***							
***COHORT STUDIES***							
Percudani [39]	1645978 All patients in Lombardy Italy with CV related Outcome in 2002 (age>=65)	Hospital Admission for CV related outcome ICD9 430--438	Moderate/Low	Yes (unmatched covariate adjustment)	2 years	2.15 %	OR (ANTIP v NU); 1.24 95% CI; 1.16-1.32
Sacchetti [40]	134488 All patients, General Practitioner Health Search database (Italy) (age>50)	Diagnosis of stroke (GPs’ medical records) ICD9: 434.9, 438.0, 342	Moderate/Moderate	Yes (covariate adjustment)	6 months	3.6 per 1000py	1 month: RR (ANTIP v NU); 12.4 95% CI; 8.4-18.1
Kleijer [41]	2448 Patients in community pharmacy practice, PHARMO Database (Netherlands) (age>50)	Hospital Admission for stroke (inc TIA) ICD9 430-436	Moderate/Low	Yes (matched: age/sex)	1 year	NA	Current use: OR (ANTIP v NU); 1.6 95% CI 1.3-2.0 0-7 days: OR (ANTIP v NU); 9.9 (5.7-17.2) 8-14 days :OR (ANTIP v NU);2.6 (1.3-5.3) 15-30 days: OR (ANTIP v NU); 2.1 (1.0-4.5) 31-90 days: OR (ANTIP v NU); 1.5 (1.0-2.2) >90 days: OR (ANTIP v NU); 1.0 (0.7-1.3)
***Studies that compared conventional antipsychotic (C) and atypical antipsychotic (A) treatment***							
***COHORT STUDIES***							
Hermann [42]	11400 All patients, population based cohort (Canada) (age>65)	Hospital Admission for stroke ICD9 430-436	Moderate/Moderate	Yes (covariate adjustment)	5 years	5.7 per 1000py	RR (RISP v CONV); 1.4 95% CI; 0.7-2.8 RR (OLA v CONV); 1.1 95% CI; 0.5-2.3
Gill [43]	32710 Dementia patients, administrative health care database (Canada) (age>=65)	Hospital Admission for ischaemic stroke ICD9 431, 434,436	Moderate/Moderate	Yes (covariate adjustment, subgroup analysis)	5 years	6.3 %	HR(ATYP v CONV); 1.01 95% CI; 0.81-1.26
Finkel [44]	18477 Dementia patients, Medicaid database (US) (age>60)	Hospital Admission for stroke ICD( 430-432, 434-436, 437.1, 437.9	Moderate/Moderate	Yes (covariate adjustment)	3 months	0.87 %	OR (OLA v RISP); 1.1 95% CI; 0.6-1.7 OR (QUE v RISP); 0.78 95% CI; 0.2-1.9 OR (HAL v RISP); 1.9 95% CI; 1.0-3.6
Percudani [39]	1645978 All patients with CV related outcome in 2002 (Italy) (age>=65)	Hospital Admission for CV related outcome ICD9 430--438	Moderate/Low	Yes (unmatched covariate adjustment)	2 years	2.37 %	OR (ATYP v CONV); 1.42 95% CI; 1.24-1.64
Wang [45]	22890 Patients in Pharmacy Assistance Contract for Elderly Program (US) (age>=65)	Hospital Admission for stroke, cerebral hemorrhagic and ischemic events	Moderate/Moderate	Yes (Propensity score adjustment, instrumental variable analysis)	180 days	Not Reported	30 days: HR (CONV v ATYP); 1.08 95% CI; 0.99-1.18 60 days: HR (CONV v ATYP) 1.10 95% CI; 1.02-1.19 180 days: HR (CONV v ATYP) 1.09 95% CI; 1.02-1.16 IV analyses not reported
Sacchetti [33]	74,162 All patients, General Practitioner Health Search database (Italy) (age>=65)	Diagnosis of stroke (GPs’ medical records) ICD9: 434.9, 438.0, 342	Moderate/Moderate	Yes (covariate adjustment, subgroup analysis)	3.5 months	47.4 per 1000py	RR (BUTY v ATYP); 1.44 95% CI; 0.55-3.76 RR (PHENO v ATYP); 2.34 95% CI; 1.01-5.41

**Table 3:** Studies on the risk of hip/femur fracture associated with antipsychotic medicines

**Study**	**Population**	**Outcome**	**Propensity for bias/Study quality**	**Procedures to minimize bias**	**Follow-up**	**Outcome Rate in reference group**	**Result**
***LEVEL III Evidence: Observational Studies***							
***Studies that compared atypical antipsychotic (ATYP) treatment to non-use (NU)***							
***COHORT STUDIES***							
Normand [46]	1286395 All patients (Canada) (age>65)	Hip fracture	Moderate/Low	Yes (covariate adjustment)	NR	NR	OR (ATYP v NU); 2.2 95% CI; 2.1-2.4
***CASE-CONTROL STUDIES***							
Liperoti [47]	1787 cases, 5606 controls Institutionalised patients (US) (age>=65)	Hospitalisation for hip fracture ICD9 820-821	Moderate/Moderate	Yes (matched on; admitted to same facility for septicemia, GI, MI)	NR	NA	OR (ATYP v NU); 1.37 95% CI; 1.11-1.69 OR (RISP v NU); 1.42 95% CI; 1.12-1.80 OR (OLA v NU); 1.34 95% CI; 0.87-2.07 OR (OTHERATYP v NU); 1.03 95% CI; 0.47-2.28
Kolanowski [35]	959 Dementia patients, health care insured on Southeast US (age>70)	Diagnosis of hip Fracture	Moderate/Low	Yes (unmatched covariate adjustment)	45 days	NA	OR (ATYP v NU); 1.47 95% CI; 0.82-2.65
Pouwels [48]	6763 cases, 26341 controls All patients, PHARMO Database (Netherlands) (age>18)	Hospitalis-ation for Hip fracture	Moderate/Low	Yes (Matched on; year of birth, sex, geographic region)	12 years	NA	OR (ATYP v NU); 0.83 95% CI; 0.42-1.65
Jalbert [49]	764 cases, 3582 controls Long stay Medicaid-eligible resident living in nursing homes with at least 20 beds (age>65)	Hospitalis-ation for Hip fracture ICD9 820	Moderate/Low	Yes (Matched; admitted to same facility, covariate adjustment)	2 years	NA	New use: OR (ATYP v NU); 1.36 95% CI; 0.95-1.94 Prevalent use: OR (ATYP v NU); 1.33 95% CI; 1.08-1.63
***SELF-CONTROLLED CASE SERIES STUDIES***							
Pratt [50]	8285, Australian Department of Veterans Affairs Veterans/spouses with hospitalization for hip fracture (Australia) (age>=65)	Hospitalis-ation for Hip fracture ICD10 S720, S721	Moderate/Moderate	Yes (within patient design)	4 years	NA	1 week: IRR (ATYP V UEXP); 2.17 95% CI; 1.54-3.06 2-8 weeks: IRR (ATYP V UEXP); 1.27 95% CI; 1.04-1.55 9-12 weeks: IRR (ATYP V UEXP); 1.23 95% CI; 0.92-1.63 >12 weeks: IRR (ATYP V UEXP); 1.43 95% CI; 1.23-1.66
***Studies that compared conventional antipsychotic (CONV) treatment to non-use (NU)***							
***CASE-CONTROL STUDIES***							
Liperoti [47]	1787 cases, 5606 controls Institutionalised patients (US) (age>=65)	Hospitalis-ation for hip fracture	Moderate	Yes (covariate adjustment and matched; on admitted to same facility for septicemia,GI,MI)	NR	NA	OR (CONV v NU); 1.35 95% CI; 1.06-1.71 OR (HALO v NU); 1.53 95% CI; 1.18-2.26 OR (OTHERCONV v NU); 1.09 95% CI; 0.78-1.52
Kolanowski [35]	959 Dementia patients, health care insured on Southeast US (age>70)	Diagnosis of hip Fracture	Moderate	Yes (covariate adjustment)	45 days	NA	OR (CONV v NU); 2.33 95% CI; 1.08-5.03
Pouwels [48]	6763 cases, 26341 controls All patients, PHARMO Database (Netherlands) (age>18)	Hospitalis-ation for Hip fracture	Moderate	Yes (Matched; year of birth, sex, geographic region)	12 years	NA	OR (CONV v NU); 1.76 95% CI; 1.48-2.08
Jalbert [49]	764 cases, 3582 controls Long stay Medicaid-eligible resident living in nursing homes with at least 20 beds (age>65)	Hospitalis-ation for Hip fracture	Moderate	Yes (Matched; admitted to same facility)	2 years	NA	Prevalent use: OR (CONV v NU); 1.28 95% CI; 0.7-2.34
***SELF-CONTROLLED CASE SERIES STUDIES***							
Pratt et al. [50]	8285, Australian Department of Veterans Affairs Veterans/spouses with hospitalization for hip fracture (Australia) (age>=65)	Hospitalis-ation for Hip fracture ICD10 S720, S721	Moderate	Yes (within patient design)	4 years	NA	1 week: IRR (CONV V UEXP); 1.04 95% CI; 0.40-2.70 2-8 weeks: IRR (CONV V UEXP); 2.23 95% CI; 1.65-3.02 9-12 weeks: IRR (CONV V UEXP); 1.79 95% CI; 1.12-2.84 >12 weeks: IRR (CONV V UEXP); 2.19 95% CI; 1.62-2.95
***Studies that compared all antipsychotics with non-use (NU)***							
***CASE-CONTROL STUDIES***							
Wang [51]	1222 cases, 4888 controls Elderly patients enrolled in Medicare as well as in the New Jersey Medicaid or Pharmaceutical Assistance to the Aged and Disabled programs (age>=65)	Hospitalis-ation for hip fracture	Moderate/Moderate	Yes (matched on; year birth and gender, covariate adjustment)	NR	NA	OR (ATYP v NU); 1.60 95% CI NR
***Studies that compared conventional antipsychotic (CONV) and atypical antipsychotic (ATYP) treatment***							
***COHORT STUDIES***							
Normand [46]	1286395 All patients, in Ontario (Canada) (age>65)	Hip fracture	Moderate/Low	Yes (covariate adjustment)	NR	NR	OR (ATYP v CONV); 0.5 95% CI; 0.4-0.5

**Table 4:** Studies on the risk of pneumonia associated with antipsychotic medicines

**Study**	**Population**	**Outcome**	**Propensity for bias/Study quality**	**Procedures to minimize bias**	**Follow-up**	**Outcome Rate in reference group**	**Result**
***LEVEL III Evidence: Observational Studies***							
***Studies that compared atypical antipsychotic (ATYP) treatment to non-use (NU)***							
***CASE-CONTROL STUDIES***							
Knol [52]	543 cases, 2163 controls All patients; no prior pneumonia, PHARMO Database (Netherlands) (age>=65)	hospital diagnosis of pneumonia ICD9 480-486, 507	Moderate/Low	Yes (unmatched covariate adjustment)	6 months	NR	OR (ATYP v NU); 3.1 95% CI; 1.9-5.1
Trifiro [53]	258 cases, 1686 controls Elderly patients from Dutch General Practice database (IPCI) patients (age>=65)	Fatal or non-fatal diagnosis of pneumonia	Moderate/Low	Yes (matched; `age/sex)	11 years	NA	Current use: OR (ATYP v PU); 2.6 95% CI; 1.5-4.6 Recent use: OR (ATYP v PU); 1.4 95% CI; 0.5-4.1
Gau [54]	194 cases, 952 controls Patients admitted to community hospital, Ontario (Canada) (age>=65)	Hospitalisation for pneumonia	Moderate/Low	Yes (matched; hospitalization for another diagnosis)	12 months	NA	OR (ATYP v NU); 2.3 95% CI; 1.2-4.2
***SELF-CONTROLLED CASE SERIES STUDIES***							
Pratt [50]	13932, Australian Department of Veterans Affairs Veterans/spouses with hospitalization for pneumonia (Australia) (age>=65)	Hospitalisation for pneumonia ICD10 J12–J18	Moderate/Moderate	Yes (within patient design)	4 years	NA	1 week:IRR (ATYP V UEXP); 1.73 95% CI; 1.31-2.29 2-8 weeks: IRR (ATYP V UEXP); 1.70 95% CI; 1.48-1.95 9-12 weeks: IRR (ATYP V UEXP); 1.67 95% CI; 1.37-2.04 >12 weeks: IRR (ATYP V UEXP); 1.70 95% CI; 1.51-1.93
***Studies that compared conventional antipsychotic (CONV) treatment to non-use (NU)***							
***CASE-CONTROL STUDIES***							
Knol [52]	543 cases, 2163 controls All patients; no prior pneumonia, PHARMO Database (Netherlands) (age>=65)	hospital diagnosis of pneumonia ICD9 480-486, 507	Moderate/Low	Yes (matched)	6 months	NA	OR (CONV v NU); 1.5 95% CI; 1.2-1.9
Trifiro[53]	258 cases, 1686 controls Elderly patients from Dutch General Practice database (IPCI) patients (age>=65)	Fatal or non-fatal diagnosis of pneumonia	Moderate/Low	Yes (matched)	11 years	NA	Current use: OR (CONV v PU); 1.8 95% CI; 1.2-2.5 Recent use: OR (CONV v PU); 1.3 95% CI; 0.8-2.0
***SELF-CONTROLLED CASE SERIES STUDIES***							
Pratt [50]	13932, Australian Department of Veterans Affairs Veterans/spouses with hospitalization for pneumonia (Australia) (age>=65)	Hospitalisation for pneumonia ICD10 J12–J18	Moderate/Moderate	Yes (within patient design)	4 years	NA	1 week: IRR (CONV V UEXP); 1.51 95% CI; 1.07, 2.14 2-8 weeks: IRR (CONV V UEXP); 1.62 95% CI; 1.37, 1.92 9-12 weeks: IRR (CONV V UEXP); 1.69 95% CI; 1.32, 2.16 >12 weeks: IRR (CONV V UEXP); 1.63 95% CI; 1.36, 1.96
***Studies that compared antipsychotic (ANTIP) treatment to non-use (NU)***							
***CASE-CONTROL STUDIES***							
Knol [52]	543 cases, 2163 controls All patients; no prior pneumonia, PHARMO Database (Netherlands) (age>=65)	hospital diagnosis of pneumonia ICD9 480-486, 507	Moderate/Low	Yes (matched)	6 months	NA	0-8 days: OR (ANTIP v NU); 4.4 95% CI; 2.9-7.2 8-14 days: OR (ANTIP v NU); 2.3 95% CI; 1.1-4.9 15-30 days: OR (ANTIP v NU); 1.9 95% CI; 1.0-3.1 31-90 days: OR (ANTIP v NU); 2.0 95% CI; 1.1-3.0 >90 days: OR (ANTIP v NU); 1.1 95% CI; 0.9-0.6
Wada [55]	121, Alzheimers patients in psychiatric hospital patients; (mean age 78.2)	pneumonia	High/Low	No (unmatched)	Not reported	Not reported	OR (ANTIP v NU); 3.13 95% CI; 1.46-6.69
***Studies that compared conventional antipsychotic (CONV) and atypical antipsychotic (ATYP) treatment***							
***COHORT STUDIES***							
Wang [45]	22890 Patients in Pharmacy Assistance Contract for Elderly Program (age>=65)	Hospital Admission for pneumonia plus prescription for an antibiotic medication	Moderate/Moderate	Yes (Propensity score adjustment, instrumental variable analysis)	180 days	NR	30 days: HR (CONV v ATYP); 1.11 95% CI; 0.76-1.63 60 days: HR (CONV v ATYP); 1.03 95% CI; 0.76-1.38 180 days: HR (CONV v ATYP); 0.84 95% CI; 0.66-1.05 IV analyses not reported
Trifiro [53]	258 cases, 1686 controls Elderly patients from Dutch General Practice database (IPCI) patients (age>=65)	Fatal or non-fatal diagnosis of pneumonia	Moderate/Low	Yes (matched)	11 years	NA	Current use: OR (ATYP V CONV); 1.48 95% CI; 0.84-2.60 Current use: OR (ATY/PHENO v BUTY); 1.86 95% CI; 1.09-3.17

### Risk of death associated with antipsychotic medicines

#### Meta-analysis and RCT evidence: studies that compared antipsychotic treatment to placebo

Meta-analyses of risperidone compared to placebo showed a non-significant 20% to 30% increased relative risk of death with short-term treatment (<12 weeks) [5,19,20]. One additional RCT found a 42% increased relative risk of death with atypical antipsychotics with extended duration of treatment [21]. Only one RCT was located that measured the risk of death associated with conventional antipsychotics compared to placebo [8]. This study, limited to 12 weeks duration, found a non-significant 68% increased relative risk of death with haloperidol [8], however, this may be due to insufficient statistical power as the number of patients in this study was small. No RCT evidence was available comparing the risk of death between the classes.

#### Observational evidence: studies that compared antipsychotic treatment to non-use

Observational cohort study evidence for atypical antipsychotics compared to non-use [22] was consistent with the longer duration RCT results [21] while a case–control study gave a much higher estimate [23]. Three observational studies found that conventional antipsychotics were associated with an increased risk of death compared to non-use [23,25,26] while one [24] case–control study found no difference in risk (Table 1).

#### Observational evidence: studies that compared conventional and atypical antipsychotic treatment

The majority of observational studies comparing the risk of death between the classes were performed using a cohort study design. Conventional antipsychotics were consistently associated with a 20-40% relative increased risk of death when compared to atypical antipsychotics [22,26-29,31,32,56]. Absolute risks, however, varied between studies according to the method employed to control for confounding. Conventional statistical methods adjusting for measured covariates suggested an increased risk over 6 months of between 2 and 3 deaths per 100 patients treated with conventional compared to atypical antipsychotics [22,27,56] while those that used an instrumental variable analysis, to adjust for unmeasured confounding, found an increased risk of between 4 and 7 deaths per 100 patients treated over 6 months [28,29] and up to 10 deaths per 100 over 12 months [31]. Only one observational cohort study [26] and one case–control study [23] found no significant difference between the classes.

### Risk of cerebrovascular events associated with antipsychotic medicines

#### Meta-analysis and RCT evidence: studies that compared antipsychotic treatment to placebo

Five meta-analyses reported a significantly increased risk of cerebrovascular events with atypical antipsychotics compared to placebo [1,4-6,32] (Table 2). When the outcome was limited to cerebrovascular events requiring hospitalisation no increased risk was observed [4,32]. No RCT evidence was located for the risk of cerebrovascular events with conventional antipsychotics or for the comparison between classes.

#### Observational evidence: studies that compared antipsychotic treatment to non-use

Observational cohort studies found similar results to the meta-analyses of atypical antipsychotics compared to placebo. Of the two cohort studies, one study, that included all diagnoses of stroke from general practitioners’ medical records, found a significantly increased risk [33], while the other, investigating the more serious outcome definition of hospitalization for stroke, found no association with atypical antipsychotics compared to non-use [34]. Two case–control studies failed to find any association between atypical antipsychotics and cerebrovascular events compared to non-use [35,36]. Two studies using a self-controlled case-series design, to adjust for unmeasured confounding, also found similar results to the RCTs when similar definitions of cerebrovascular events were considered. One study found an increased risk of stroke as diagnosed through general practitioners’ medical records for up to 70 days after initiation of atypical antipsychotics [37] while the other found no increased risk of hospitalization for stroke after initiation of atypical antipsychotics [38].

The strongest available evidence for the risk of cerebrovascular events with conventional antipsychotics was from observational cohort studies. One study found a significantly increased risk of stroke as diagnosed in general practitioners’ medical records [33] while the other found no increased risk of hospital admissions for cerebrovascular events [34] (Table 2). Two case–control studies failed to find any association between conventional antipsychotics and cerebrovascular events [35,36]. One self-controlled case-series study found an increased risk of stroke, as diagnosed in GPs’ medical records, with conventional antipsychotic initiation compared to non-use which persisted up to 140 days after treatment initiation [37]. Another self-controlled case-series study found that the risk of hospitalization for stroke was increased in the first week only after initiation of antispychotics [38].

A temporal association between antipsychotics and cerebrovascular events was identified in observational studies. Three studies, all using different study designs, found that the risk associated with antipsychotic initiation was highest immediately following treatment initiation but returned to base-line with longer term treatment [37,40,41].

#### Observational evidence: studies that compared conventional and atypical antipsychotic treatment

Six cohort studies compared the risk of stroke between the classes with conflicting results. Three studies found equivalent risk between the classes [42-44]. One study found a reduced risk with conventional compared to atypical [39] while two found an increased risk [33,45] with conventional compared to atypical antipsychotics (Table 2). An instrumental variable analysis found an increased risk of stroke with conventional antispychotics, however, the numerical results of this analysis were not presented [45].

### Risk of hip fracture associated with antipsychotic medicines

#### Meta-analysis and RCT evidence: studies that compared antipsychotic treatment to placebo

No RCT evidence for the risk of hip fracture associated with either conventional or atypical antipsychotics was located.

#### Observational evidence: studies that compared antipsychotic treatment to non-use

Only one observational study employing a cohort design [46] was located that investigated the association between antipsychotics and hip fracture. This study found a 2.2 times excess risk of hip fracture with atypical antispychotics compared to non use. Of the four case–control studies, one found an increased risk of hip fracture with atypical antipsychotics compared to non-use[47], two studies found no increased risk [35,48] and one study found equivalent risk in new users but increased risk in prevalent users [49]. A self-controlled case-series study [50] found a significantly increased risk of hospitalization for hip fracture with atypical antipsychotics which was highest in the first week after initiation. The risk declined with increased duration of treatment and remained significantly raised by over 40% with long-term treatment.

Conventional antipsychotics were associated with a significantly increased risk of hip fracture in three case–control studies [35,47,48], while one case–control study found no increased risk [49]. A self-controlled case-series study [50] found a significantly increased risk of hip fracture with conventional antipsychotics after 1 week continuous treatment.

#### Observational evidence: studies that compared atypical and conventional antipsychotic treatment

Only one cohort study [46] was identified that directly compared the risk of hip fracture between the classes of antipsychotics. This study found a significantly increased risk of hip fracture with conventional antipsychotics compared to atypical antipsychotics [46] (Table 3).

### Risk of pneumonia associated with antipsychotic medicines

#### Meta-analysis and RCT evidence: studies that compared antipsychotic treatment to placebo

No RCT evidence for the risk of pneumonia associated with either atypical or conventional antipsychotics was located.

#### Observational evidence: studies that compared antipsychotic treatment to non-use

Observational evidence for the risk of pneumonia with atypical antispychotics compared to non-use was limited to three case–control studies [52-54] and one self controlled case-series study [50]. The case–control studies found a 2–3 times increased risk of pneumonia [52-54] (Table 4). A self controlled case-series study found a 70% increased risk of pneumonia after initiation of atypical antipsychotics [50].

Two case–control studies found a significantly increased risk of pneumonia with conventional antipsychotics[52,53] and one self-controlled case-series study found a 60% increased risk compared to non-exposure.

Two case–control studies of all antipsychotics combined identified a significantly increased risk of pneumonia associated with treatment [52,55]. One study found that the risk was highest in the first week of treatment and returned to base-line levels after more than 90 days treatment [52].

#### Observational evidence: studies that compared atypical and conventional antipsychotic treatment

Two studies were located comparing the risk of pneumonia between the classes [46,54]. Both studies found no difference in the risk of pneumonia between the classes [45] (Table 4).

## Discussion

This review included 44 studies that evaluated the risk of either death, cerebrovascular events, hip fracture or pneumonia associated with antipsychotic prescribing in the elderly. We found that observational evidence appears to support the findings from RCTs, where available, however the magnitude risk differed according to the methods used to control for confounding.

RCT evidence for atypical antipsychotics showed an absolute increase of 1 extra death per 100 people treated. Collectively, observational evidence showed that conventional antipsychotics were associated with a greater risk of death than the atypical antipsychotics, however, the estimates of absolute risk differed between studies. Risk estimates from covariate adjusted cohort studies ranged from 2 to 3 extra deaths per 100 patients treated with conventional compared to atypical antipsychotics over 6 months while instrumental variable analysis estimates ranged from 4 and 7 extra deaths per 100 patients over 6 months. These discrepancies suggest that unmeasured confounding may have contributed to an underestimate of risk in the traditional cohort studies. Many of the cohort studies also employed propensity score methods to adjust for confounding either by use of propensity score matching [22,26] or numerical adjustment by propensity score quintiles [28,29,31]. In these latter studies propensity score adjustment estimates were consistent with multivariate adjusted results while instrumental variable analyses were marginally different suggesting some residual unmeasured confounding in the propensity score and multivariate adjusted analyses. Additionally studies have undertaken sensitivity analyses to rule out potential bias from unmeasured confounding which revealed that only very strong unmeasured confounders would explain the observed increased mortality association with conventional antipsychotic use, if in fact, the observed difference was not true [22,28].

RCT evidence of the risk of cerebrovascular events was limited to the atypical antipsychotics and demonstrated an increased risk of all cerebrovascular events[1] but not serious strokes requiring hospitalization [4,33]. Cerebrovascular events were the most studied and reported adverse event in observational studies of antipsychotics compared to non-use, however, the definition of this outcome was not consistent between studies. In general, cohort studies reported negative associations when investigating cerebrovascular hospitalisation events and positive associations when investigating outcomes defined as a diagnosis of all cerebrovascular events from general practitioners’ medical records which supports the findings from available RCTs. In contrast, case–control studies failed to find statistically significant results for either outcome definition. Case–control studies often employ techniques to minimise possible bias, such as matching or numerical adjustment for potential confounders, however, studies of this type may still be subject to unmeasured confounding [57]. Self-controlled case-series studies found similar results to RCT evidence when similar definition of cerebrovascular events were used which suggests that this technique may be a reliable design for the investigation of adverse events not previously detected in RCTs. One of the advantages of the self-controlled case-series design is that it controls implicitly for patient-specific confounders that do not vary over time. This means that it is not necessary to adjust for variables such as sex, frailty or other risk factors that are constant over time. However, a limitation of this approach is that it is unable to adjust for changes in prescribing due to rapid changes in underlying disease severity [17]. Observational cohort studies comparing the antipsychotic classes have used consistent definitions of outcome, specifically, hospitalisation for stroke, however, results vary according to the methods employed to adjust for differences between treatment groups. Of the 6 studies that used numerical covariate adjustment, three found no difference in risk [42-44] between the classes, one found a reduced risk [39], while two found an increased risk [33,45] with conventional compared to atypical antispychotics. One study [45], used an instrumental variable method to adjust for unmeasured confounding. This study found a 10% increased risk of stroke with conventional compared to atypical antipsychotics but only after 60 days treatment, however, the instrumental variable estimates were not presented and the prevalence of cerebrovascular disease at baseline in the studied population, described in a separate paper, was high [29].

While no RCT data for the risk of hospitalisation for hip fracture could be located, a Cochrane review [1] found that risperidone may be associated with an increased risk of falls in the elderly which suggests that an increased risk of hip fracture may also be likely. Observational studies reported an increased risk of hip fracture with both classes compared to non-use and this risk may increase with increasing duration of therapy. A study using a self-controlled case-series design [50], found an increased risk of hospitalization for hip fracture for both conventional and atypical antipsychotics. It is unclear whether the risk of hip fracture differs between the classes. Only one cohort study [46] investigated the comparative risk of hip fracture between the classes finding an increased risk with conventional antipsychotics.

Meta-analyses [19-50,52-58] of randomized controlled trials found that one of the major causes of death associated with atypical antipsychotics was pneumonia. Few observational studies, however, have investigated the risk of pneumonia associated with antipsychotics in elderly patients, and most have used a case–control design. An increased risk of pneumonia was found with both classes [52,55] compared to non-use or previous use [53]. One case–control study identified that the risk appeared to be highest in the first week of treatment but returned to baseline after 90 days [52]. A study using a self-controlled case-series design [50] also found and increased risk of hospitalization for pneumonia for both conventional and atypical antipsychotics. The risk of pneumonia was similar between the classes [46,54]. One study, which used an instrumental variable analysis, found no difference in the risk of pneumonia between conventional and atypical antipsychotics [45].

As in any systematic review, publication bias is a potential limitation of this study. It is possible that only positive observational study findings may have been published. In particular, the majority of published studies investigating hip fracture and pneumonia associated with antipsychotics reported significantly increased risks with treatment compared to non-use. Additionally, our search criteria specified that only those study designs that resulted in standard effect estimates were included for comparison purposes. We therefore excluded case reports and studies that used designs such as the self-controlled cohort analysis [59]. We only included outcomes that could be consistently defined to limit heterogeneity across studies. The endpoints for this study, cerebrovascular events, hip fracture and pneumonia, were chosen because we believed that patients were likely to present to hospital for these conditions, and therefore were outcomes that would most likely be available in observational studies using administrative claims databases. The application of methods such as the self controlled case-series and instrumental variable analyses have evolved mainly to address unmeasured confounding due to the lack of clinical information in claims databases. Antipsychotics have also been associated with other adverse events such as deep vein thrombosis, diabetes onset and heat stroke which were not included in this review.

The aim of this review was to explore the effects of study design on the results of observational studies of antipsychotics. To do this we simplified our comparisons to with-in class comparisons and we have not considered differences according to individual products, dose or effect modifiers. The majority of published observational studies have provided comparisons between classes, however, where available we have presented the results by individual products and by dose. Besides study design, many other factors may have contributed to the differences observed among observational studies. These include differences in populations studied, in terms of drug utilization patterns and health care settings, differences in the definition of outcome and variations in the number and type of confounders that may have been included in the analysis. We have attempted to limit the impact of these effects by considering the effects of antipsychotics in the elderly only and by limiting our events to serious adverse events potentially requiring hospitalization. In the case of cerebrovascular events we considered cerebrovascular events separately to hospitalizations for stroke as these were the two events most often considered in observational studies.

Where possible we compared available meta-analyses and randomised controlled trial evidence with the results of observational studies, however, experimental evidence was typically generated prior to when many of the observational studies were performed and may have included different patient populations. RCT evidence is generally considered of higher quality than observational studies,[18] however, there are many examples where RCTs on the same clinical topic have produced conflicting results [60]. High quality observational studies, whether cohort or case–control designs, have consistently found similar results to RCTs [60].

## Conclusions

This review has identified that the harms associated with antipsychotics in the elderly are under reported in published RCTs and the risks of treatment may not be limited to death and cerebrovascular events. Collectively, evidence suggests that atypical antipsychotics are associated with an increased risk of death, cerebrovascular events, hip fracture and pneumonia. Based on RCT evidence [5,6,8,61-63] the number needed to treat with risperidone to show clinical benefit ranges from 3 to 9 patients over a 12 week period (Table 5). This means that for every 100 patients treated with risperidone we would expect between 8 and 33 patients to receive any clinical improvement in symptoms of aggression or psychosis. The risk-benefit ratio suggests that there will be 1 excess death for every 11 to 33 person helped with these medicines [19], 1 excess hospitalization for hip fracture for every 4 to 12 patients helped and 1 excess hospitalization for pneumonia for every 2 to 5 patients helped [50]. Considering the modest improvement in terms of efficacy and the increased risk of mortality associated with hip fracture and pneumonia, the risks associated with antipsychotics may now outweigh their benefit. In the absence of RCT data, good quality observational studies will be required, that employ appropriate study designs that are robust towards unmeasured confounding, to clarify the risks of antipsychotic use in the elderly.

**Table 5:** Efficacy of atypical antipsychotics in elderly patients with dementia: Number needed to treat

***Study***		***Study Design***	***Follow-up***	***Risperidone n/N (%)***	***Placebo n/N (%)***	***RD***	***NNT***
***Clinical End Point***							
**>50% Improvement in Behave-AD**^**1**^**total score**							
	Katz [62]	Double-blind placebo controlled RCT (n=625)	12 weeks	(45%)	(33%)	12%	8
	Schneider [6]	Meta Analysis of 3 studies (n=1001)	12 weeks	266/574 (46%)	139/427 (33%)	14%	7.4
**>30% Improvement in Behave-AD**^**1**^**total score**							
	DeDeyn [8]	Double-blind placebo controlled RCT (n=344)	12 weeks	(72%)	(61%)	11%	9
**CGI-C**^**2**^** (much/very much improved)**							
	Brodaty [61]	Double-blind placebo controlled RCT (n=93)	12 weeks	27/46 (59%)	12/47 (26%)	33%	3.3
	Schneider [6]	Meta Analysis of 2 studies (n=717)	8-12 weeks	227/351 (65%)	175/366 (48%)	17%	6
	Katz [5]	Meta Analysis of 4 studies (n=889)	End point	(28%)	(17%)	11%	9
	Sultzer [63]	Double-blind placebo controlled RCT (n=421)	12 weeks	(61%)	(40%)	21%	5

^1^ BEHAVE-AD: Behaviour Pathology in Alzheimer’s Disease Rating Scale.

^2^ CGI-C: Clinical Global Impression of Change.

## Competing interests

The authors have no competing interests to declare.

## Authors’ contributions

NP and ER carried out the identification of studies to be included in this review. All authors participated in the design and interpretation of the study. All authors contributed to the drafting of the manuscript and have approved the final manuscript.

## Pre-publication history

The pre-publication history for this paper can be accessed here:

http://www.biomedcentral.com/1471-2288/12/72/prepub

## Supplementary Material

Additional file 1 PRISMA 2009 Checklist. Click here for file
